# Joint family consultations for psychiatric inpatients with severe eating disorders transitioning to adulthood: psychoanalytic design of a therapeutic setting

**DOI:** 10.3389/fpsyt.2023.1270499

**Published:** 2023-10-09

**Authors:** Olivier Putois, Myriam Riegert, Nadine Bahi, Joël Pires, Manuella De Luca

**Affiliations:** ^1^SuLiSoM UR 3071, Faculté de Psychologie, Université de Strasbourg, Strasbourg, France; ^2^Service de Psychiatrie, Santé Mentale et Addictologie, Hôpitaux Universitaires de Strasbourg, Strasbourg, France; ^3^Institut d’Immunologie et d’Hématologie, Institut Thématique Interdisciplinaire TRANSPLANTEX NG, Université de Strasbourg, Strasbourg, France; ^4^Institut Contemporain de l’Enfance, Paris, France; ^5^PSInstitut, Strasbourg, France; ^6^PCPP UR 4056, Institut de Psychologie, Université de Paris, Paris, France

**Keywords:** inscription, therapeutic setting, eating disorders, family, denial, model, psychosomatic, psychoanalysis

## Abstract

While family work is acknowledged as relevant in the care of eating disorders (EDs), not much literature has explored it in the period of transition from adolescence to young adulthood (16–18 to 30 y.o.). Yet this period is of significant importance in the prognosis and evolution of EDs; but its particular stakes require specific therapeutic settings–especially for inpatient EDs. In this paper, we start from the paradoxical observation that some families refuse this type of work in its usual form, with a family-dedicated therapist, and require to only exchange with the psychiatrist in charge of the treatment plan. We use a psychosomatic-informed psychoanalytic approach to shed light on this refusal as a latent denial of the contribution of family dynamics to the current symptom, and an unconscious tendency to stick to a dependency-laden family scheme. We then explain the conception of a specifically dedicated therapeutic setting, designed to address this specific type of resistance, offered to families as a therapeutic compromise designed to give them a specific position in the care of their child. In our joint therapeutic consultations, family dynamics are addressed on the basis of exchanges regarding treatment and in particular feeding. While such exchanges start from medical considerations, the therapeutic couple (psychiatrist-psychologist) uses them to address the parent and patient expectations underlying the symptom. We propose to call this act “inscription”; it enables a separation from the underlying dependency-oriented family scheme, while stressing the importance to care for associated parental anxieties.

## 1. Introduction

### 1.1. Eating disorders in late adolescence and young adulthood: complexity and family care

Eating disorders (EDs) encompass a vast array of complex psychiatric pathologies present in adolescents and young adults, such as binge eating, *anorexia nervosa*, etc. The complexity of these pathologies comes from their notoriously multi-factorial nature – factors encompass culture, family, somatic and biological conditions, and psychiatric comorbidities (1). The absence of a univocal causality requires a multi-faceted approach to care.

Amongst these factors, therapeutic paradigms have stressed the need to address family dynamics, thereby including families to the treatment plan [cf. e.g., Rienecke and Le Grange (2)]. Offering them punctual psychotherapeutic consultations or regular therapy sessions is standard practice for adolescent inpatient EDs in French hospital Units (3, 4).

Less literature has stressed the importance of family work in the transition to young adulthood (16–18 to 30 y.o.). Yet we believe, like (5–7), that this period is of importance in the prognosis and evolution of EDs, and requires specific therapeutic settings. This is especially the case for serious forms, requiring inpatient care.

In this respect, we have been struck by a recurring feature encountered in many inpatient clinical situations in this age range (the vast majority of EDs in the adult Psychiatry Unit at Strasbourg University Hospital). A significant number of families refuse to explore the family dimension of the symptoms in a dedicated family consultation with a psychologist, in spite of the seriousness of their child’s condition–which leads others to accept every therapeutic option. Instead, when proposed such a (state-funded) dedicated consultation, they say that it is not that they do not want it, but that since it is now the hospital’s job to cure their child, they see no reason to discuss the situation and its family effect.

Drawing on psychoanalysis – an acknowledged therapeutic paradigm in EDs [e.g., Thompson-Brenner et al. (1)], we consider this refusal as a form of specific family resistance against the anxiety induced by the offer to explore family dynamics, and *a fortiori* subsequent family changes. To work with this subset of families, we devised a specific therapeutic setting adapted to this resistance: that of joint family consultations (JFCs), jointly led by a psychiatrist and a psychologist-psychoanalyst. The goal of this paper is to sketch out the design, therapeutic stakes and effects of this specific setting and its use, by explaining how these recurrent clinical situations led us to model it. The data presented in this paper did not need ethical clearance, as it was a secondary account of our experiences in healthcare.

## 2. Institutional context

In late adolescence, EDs are triggered by the challenges of approaching adulthood: leaving home, facing important choices (e.g., higher education), etc. This differs from earlier adolescence: what is now at stake is the possibility to withstand the perspective of psychologically separating oneself from one’s family environment and become autonomous. This has direct implications for ED inpatient care: caretakers no longer envision hospital stays as temporary breaks from family life, as they mostly are during adolescence. Hospital stays in our unit are conceived as an intermediate step before the beginning of (young) adult life, where patients separate themselves from the hospital and establish a new relationship with their families to start an adult life.

But when patients initially come to the hospital after a consultation request, and meet with the psychiatrist overseeing the treatment plan (MR), medical concerns are in the forefront–especially body mass index (BMI) issues. They are understandably the main focus of both patients, families and caretakers. Yet, as mentioned above, a growing consensus is that efficient EDs care requires family work: thus, in the case of potential inpatient admission, the psychiatrist quickly proposes to meet with the parents. Most of the time, she then offers them to plan a consultation (generally with the patient) with the unit’s dedicated psychologist/family therapist (OP), to discuss the family effects of the situation; such consultations typically explore the difficulties associated with becoming psychically autonomous, for both families and patients. This offer is presented as an addition to all standard inpatient care: daily psychotherapeutic sessions with a psychiatrist (other than the head of ED Unit), exchanges with another psychiatrist about treatment adjustment (medication, somatic follow-up, stay duration, etc.), and a wide range of institutional care: individual or group dietary support, physical and bodywork and somatic therapies, group psychotherapy, dance lessons, etc. In addition, we have the opportunity to interact with the hospital’s Clinical Nutrition Unit. Therefore, all therapeutic effects of family work take place within a dense web of activities; and it is never meant to replace individual psychotherapy, but it facilitates it (more in Conclusion).

## 3. From manifest refusal to latent common denial

But a significant subset of parents refuse to meet with the unit’s family therapist (OP) to discuss the family effects of the situation: it is not that they do not want it, but since it is now the hospital’s job to take care of their child, they see no reason to come discuss the situation and its family effect. This is particularly interesting, since EDs – especially *anorexia nervosa*, with its extremely slim bodies – tend to put many families in a constant state of preoccupation (8): quite a few immediately and gratefully accept dedicated consultations with a specialized psychologist. How are we to understand this?

While this clinical observation matches Minuchin et al.’s (9) psychosomatic remarks on change-averse families in *anorexia nervosa*, we understand it exclusively through a psychoanalytic lens: as a resistance of the family group (parents + inpatient + siblings, potentially), expressing a mechanism of denial of the family dimension at play in EDs. This refusal, and the subsequent claim that it is now the hospital’s task (and not theirs anymore) to take care of the patient (including feeding them), should be understood as an unconscious projection, onto the Psychiatry Unit, of the family’s representation of what it is to take care of someone. Considering how families tell us that they are now solely concerned with how the Psychiatry Unit takes care of their child, we contend that there is something specific in their unconscious representation of such care – and most likely, that it is a relation of exclusive dependency [cf. Corcos (3)], whereby the child would receive from the parents everything they need. While often feeling hurt because they believe they have failed to provide their child with everything they need, parents nonetheless transpose the same exclusive relational scheme onto the caregiving team, without realizing that it is its exclusive character that should be re-examined and questioned: exclusive dependency is both impossible in reality, and an obstacle to separation and individual self-realization (This accounts for the two extremes often to be found in these families: those who try and provide everything, and those who renounce because it looks impossible – both share a representation of exclusive care).

This understanding relies on the psychoanalytic concept of denial, first laid out extensively by Freud in 1938 (10), subsequently elaborated by Klein (11) and Bion (12–14), and recently developed by Ogden (15) under the name of projective disavowal. Gabbard (16) stresses its relevance and sums it up like this: denial, or disavowal, is an active process whereby denied representations or affects are projected onto someone else, in actual interactions, part of which take place at an unconscious level. The task of the therapist is to “process and contain them,” i.e., understand them as such, in order to signify to the patient (or the group) what they have thus unconsciously set aside.

We believe that when refusing a dedicated EDs consultation with the psychologist, families project onto the psychiatrist who offers it their unconscious representation of caring for a child *qua* exclusive dependency. Hence the shift of the burden of care, so to speak: it was all on the family, and it is now all on the hospital.

To specify the “interpersonal pressure” exerted on the psychiatrist, and what “process and contain” means, we expand on Braunschweig and Fain’s (17) concept of “community of denial” [cf. also (18)], following recent work (19, 20). Braunschweig and Fain (17) stressed that in situations of denial, projection is an attempt to persuade the therapist to adopt one’s perspective and jointly disavow the representations one seeks to ignore (“common” denial). In essence, these families tell the psychiatrist (a doctor) that they agree to come and see her to receive medical information about the treatment course, as long as family dynamics are not brought forward as a therapeutic dimension *per se*. This unconscious invitation to denial generally emanates from the whole family (the child partakes in it), but is most often expressed by parents. Therefore, to “process and contain” it means that the psychiatrist, in charge of both mental and physical health, needs to acknowledge the denied representation of parental care that underlies it – one of exclusiveness and dependency, which she is encouraged to take on as a parental substitute. In terms of psychoanalytic technique, she needs to acknowledge a specific form of emerging transference emanating from the whole family group and especially the parents, whereby she is envisioned as a potential accomplice of a specific, unquestioned understanding of care. This emerging transference thus represents the main therapeutic indication for our specific therapeutic setting.

## 4. Offering a joint therapeutic setting

Following a line of thought first laid out by P. Marty and M. Fain, which OP’s work develops, we decided that to preserve potential psychotherapeutic effects, the psychiatrist overseeing the treatment plan should offer a creative compromise adapted to the families’ initial resistance. On the one hand, her stance should implicitly acknowledge families’ denial-based resistance to address their relational dynamics. On the other, she needs to communicate that it is not possible to fully separate medical/somatic work from family dynamics: there can be no “splitting” between the two (10, 16, 17) since family dynamics are affected by EDs, and affect them in return. On the basis of OP’s previous research, we devised a specific therapeutic offer: that of a joint therapeutic setting, with the psychiatrist (MR) and the unit’s specialized family therapist (OP). This offer’s goal is to communicate that optimal care for the patient will respect their resistance to some extent (the psychiatrist’s presence means that it will still be possible to talk about medical care, incl. feeding/eating); but that making care optimal would *also* require to explore family feelings (this is embodied by the presence of the family therapist).

The psychiatrist tries to enact an “inscription” [cf. e.g., Chervet (21, 22)]. In our use of the term, in institutional and family group dynamics, “inscription” refers to an intervention going, in part, against families’ resistance, by shedding light on a representation that they expect the psychiatrist to deny along with them – i.e., their exclusive representation of parental care. In therapeutic technique, this inscription amounts to a specific handling of the family’s emerging transference, whereby the psychiatrist is cathected as a parental substitute mirroring their representation of care. While they expect her to mirror this representation, she draws on this transferential expectation to surprise them by expressing that she cannot take this role alone, and instead needs someone to help her. She thus communicates two elements that embody a different caregiving stance:

(1) there should be no individual/exclusive caretaker(s). This is a dismissal of their projected expectation of an almighty caretaker/caretaking team.
(2) care is not only about manifest behavioral parameters (weight, caloric intake, etc.). From the Unit’s point of view, care includes more psychological elements: at least, taking into account how everyone feels about the situation. Underlying this is a technical premise: elements regarding medical care can be handled as a therapeutic medium to address family dynamics.

The goal is a re-mobilization of the family: caregivers are not almighty, they need the parents to contribute to the care, as it involves a psychological parameter – their relation to their child. That is, ED treatment is a 3-tier challenge: patient/family/caretakers. This first inscription leaves a trace potentiating further therapist interventions during joint consultations, by delineating an alternative, non-exclusive model of care which does not require the patient to remain dependent upon the caregiver.

## 5. The technique of joint family consultations: inscription as separation

Offering such a compromise-based therapeutic setting is not psychoanalytically orthodox, since it leaves room to discuss medical and eating-related preoccupations while seeking to address elements pertaining to intra- and interpersonal psychological dynamics. It could thus at first sight be understood as feeding families’ resistance. We believe that on the contrary, in cases of strong family denial, it helps induce authentic therapeutic effects by handling their specific transference (projected expectation of an almighty caretaker). Yet, this should not be confused with family therapy *per se*, which is more constrained in terms of rhythm, participants and therapeutic aim [cf. e.g., Robert (23) or Berger (24)] since it is focused on restructuring relationships within the family – while our consultations are more punctual and seek to increase individual autonomy with respect to family dynamics.

In transition to adulthood, the therapeutic aim of these JFCs is to enable parents and patients to withstand the patient’s separation and self-realization. Such self-realization requires, as a necessary condition, feeding oneself well enough (not too little, not too much, etc.): that is, (1) not to depend on an external figure to regulate or even control eating behavior, as well as (2) accept to feed on something which does not come from one’s parents. Thus, leaving room for families and especially parents to express their feelings about the concrete care of their child is a springboard to explore how families can tolerate transitioning out of a relationship of exclusive dependency. But this requires that psychological separation be explicitly expressed in the course of a therapeutic exchange or sequence, which will echo the first inscription carried out by the psychiatrist.

For the sake of brevity, we give one frequent example: parents often tell the psychiatrist they are preoccupied because in the Unit, their child will not be offered the food they are used to; surely they will not like it as much, so how are they to be cured from their disorder? This manifest question is underlain by latent issues, through what psychoanalysis calls unconscious overdetermination. For example, in the later course of family consultations, some families admit they are concerned their child could be poisoned by what comes from the outside, while others say they are afraid the child could not control him/herself (and would always need someone to regulate their intake, like an infant). Sometimes, they say: the child is not gaining enough weight–you must feed them the wrong way. Both exemplify unconscious motives to maintain an exclusive relationship with the family: they are types of an exclusive, dependency-laden family relationship, split off from the explicit concern about feeding, and unconsciously projected onto the child who, in return, identifies with them. It is these projections that JFCs wish to disentangle, by showing that the child’s behavior and discourse to some extent echoes that of parents, and that both play a part in the current symptom.

Drawing on the presence of the psychiatrist, parents thus use manifest issues to express their rivalry with the Unit. These exchanges call for an inscription of the main psychological stake underlying EDs and their medical care: psychological separation between parents and child/patient – that is, a separation of individual perspectives through an explicit re-attribution to parents of what they project onto the child, and vice-versa. From a technical point of view, in the aforementioned type of situations, the therapeutic couple (psychiatrist-psychologist) often proceeds as follows. The psychiatrist shifts her stance out of the medical level, and stresses that from the perspective of the treatment plan, medical parameters (BMI etc.) are but one level of information, and that the stakes are more general and revolve around their child’s personal evolution, and how they feel about it. This paves the way for the psychologist to mention that maybe their worry about feeding means that it is they who suffer from being separated from their child – a suffering which they project onto him/her, and to which the child might conform to show them how irreplaceable they are, thereby maintaining dependency.

In these situations, we respectfully tell parents that their anxieties should be discussed without any form of prejudice, as they play a role in the situation, which cannot therefore be fully ascribed to the child. Upon hearing this, the patient is put in a position where s/he is both relieved of parental projections (s/he can refer to this later), while parental separation anxieties can be acknowledged; at the same time, s/he becomes authorized by a third party (the therapeutic couple) to express personal heretofore unexpressed concerns, while evolving away from such projections.

## 6. Conclusion: deferred and distributed therapeutic effects

The trace of this 2-step inscription often appears in a deferred manner, when the Unit’s individual psychotherapists tell us in a subsequent staff meeting that the patient now expresses new desires (with which e.g., their parents might not be happy); or that, more generally, they associate more freely about future perspectives. Of course, in other instances, we see that the patient’s relation to their parents evolves, often by witnessing how previous inscriptions affect their exchanges.

Finally, such a therapeutic setting is devised as a potential preparation for, or adjunction to, individual therapy; but by no means as a substitution. Indeed, some parents start a therapy after JFCs, when realizing their child’s symptom echoes their concerns. We even sometimes decide to see parents without the patient when we feel they need a temporary dedicated space to express concerns that should remain out of the child’s conscious awareness, to help them transition toward individual therapeutic work.

## Data availability statement

The original contributions presented in this study are included in this article/supplementary material, further inquiries can be directed to the corresponding author/s.

## Author contributions

OP: Conceptualization, Investigation, Methodology, Supervision, Writing – original draft, Writing – review & editing. MR: Conceptualization, Investigation, Methodology, Writing – original draft, Writing – review & editing. NB: Conceptualization, Writing – review & editing. JP: Conceptualization, Methodology, Writing – review & editing. MD: Conceptualization, Methodology, Writing – review & editing.
